# The impact of long COVID on physical and cardiorespiratory parameters: A systematic review

**DOI:** 10.1371/journal.pone.0318707

**Published:** 2025-06-04

**Authors:** Imane Salmam, Marc-Olivier Dubé, Imane Zahouani, Alexis Ramos, François Desmeules, Krista L. Best, Jean-Sébastien Roy

**Affiliations:** 1 Center for Interdisciplinary Research in Rehabilitation and Social Integration (Cirris), Quebec City, Quebec, Canada; 2 School of Rehabilitation Sciences, Faculty of Medicine, Université Laval, Quebec City, Quebec, Canada; 3 La Trobe Sport and Exercise Medicine Research Center, La Trobe University, Melbourne, Victoria, Australia; 4 Orthopedic Clinical Research Unit, Maisonneuve-Rosemont Hospital Research Center, University of Montreal Affiliated Research Center, Montreal, Quebec, Canada; 5 École Universitaire de Kinésithérapie Centre-Val de Loire, Université d’Orléans, Orléans, France; 6 School of Rehabilitation, Faculty of Medicine, University of Montreal, Montreal, Quebec, Canada; University of Sharjah, UNITED ARAB EMIRATES

## Abstract

**Background:**

Since the emergence of COVID-19, millions worldwide have continued to experience persistent symptoms months after infection. Among these, physical and cardiorespiratory impairments are frequently reported, but remain poorly understood. This systematic review aimed to identify and synthesize evidence regarding physical and cardiorespiratory impairments in individuals with long COVID, defined as symptoms persisting for at least three months post-infection.

**Methods and findings:**

A structured search was conducted across the MEDLINE, Embase, CINAHL, and Web of Science databases to identify cross-sectional and longitudinal cohort studies on physical and cardiorespiratory deficits in adults with long COVID. Twenty-two studies involving 3,041 adults with long COVID were included. Critical appraisal using the JBI-APT indicated that most studies had clear inclusion criteria (17/22), well-defined study populations (17/22), and valid exposure measurements (16/22), though confounding factors were often unaddressed (9/22 unclear or not reported). Findings indicate that while adults with long COVID displayed normal pulmonary function at rest, including Forced Vital Capacity (FVC), Forced Expiratory Volume (FEV_1_), Total Lung Capacity (TLC), and resting Arterial oxygen saturation (SpO_2_)_,_ significant impairments in exercise capacity were identified. Notably, all studies assessing the 6-minute walk test (6MWT) reported reduced distances, consistently falling below the 50^th^ percentile of normative values. Additionally, VO₂_peak_ was decreased in most studies (7/10), falling below 80% of the predicted value, indicating impaired aerobic capacity. Lower Diffusing Capacity of the Lungs for Carbon Monoxide (DLCO) values were observed in three out of six studies, with values below 75% of predicted, suggesting impaired gas exchange efficiency during exertion.

**Conclusion:**

Despite preserved resting lung function, these findings highlight significant physical deconditioning in Long COVID adults, with substantial reduction in exercise capacity. Routine assessments should include more sensitive measures, such as the 6MWT and VO₂peak, to detect subtle exercise limitations, even in patients with normal resting SpO₂, to better inform rehabilitation interventions.

## Introduction

Since the onset of the COVID-19 pandemic in 2020, nearly 776 million people globally have officially tested positive for COVID-19 [1]. However, the actual number is likely much higher due to unreported cases among asymptomatic individuals or those who were not tested. The duration and severity of symptoms vary considerably among adults infected with COVID-19. While most individuals experience a rapid recovery, approximately 10–20% develop mid- to long-term symptoms following their infection [2], a condition referred to as “Long COVID syndrome” or “post-acute sequelae of SARS-CoV-2 infection”. The World Health Organization (WHO) defines long COVID as the persistent of symptoms lasting beyond three months after the initial infection [2]. The most commonly reported persistent symptoms include fatigue, shortness of breath, muscle weakness, joint pain, headaches, as well as cognitive and physical impairments [3,4]. Beyond the physical symptoms, individuals with long COVID often endure significant psychological distress, such as anxiety, depression, and stress, exacerbating the challenges of their medical condition [5,6].

The wide range of symptoms experienced by adults with long COVID complicates efforts to fully understand this condition. Regardless of symptoms type, individuals with long COVID often face reduced participation in daily and social activities, negatively impacting their quality of life [7,8], and increasing the need for medical consultations and healthcare services [9–11]. Individuals with long COVID have been shown to average 30 healthcare visits per year and incur 43% higher annual healthcare costs compared to unaffected individuals [12]. This places a greater strain on healthcare systems worldwide and contributes to substantial financial burden due to reduced work capacity, long-term disability, and lost productivity [13]. In the United States alone, the annual societal cost of long COVID is estimated to range from $2 to $30 billion, with productivity losses accounting for over 90% of this burden [13]. On a worldwide scale, a recent study estimates the cumulative global incidence of long COVID at approximately 400 million people, with an annual economic impact of $1,000 billion, equivalent to 1% of the global economy [14].

Although millions have been affected by long COVID since the pandemic began in 2020 [15], the associated physical and cardiorespiratory impairments remain not fully understood. Long COVID is recognized as a complex, systemic disorder that can potentially affect nearly every organ system, leading to severe disability [14]. Several interrelated pathophysiological mechanisms are believed to contribute to its persistent symptoms. Chronic inflammation [16–18] and immune dysregulation [19–21], including prolonged cytokine activation and altered immune responses, may play a key role. Endothelial dysfunction, leading to vascular inflammation and microthrombosis, could impair oxygen and nutrient delivery to tissues, potentially explaining symptoms like fatigue [22–25]. Additionally, metabolic alterations, such as mitochondrial dysfunction and disrupted glucose metabolism, have also been reported in individuals with long COVID [26,27]. These mechanisms may act collectively or independently, leading to the wide range of persistent symptoms observed in long COVID [28,29].

Previous studies have highlighted the complexity of long COVID, reporting both normal and impaired pulmonary function, alongside reduced exercise capacity [30–32]. However, the variability in these findings calls for a comprehensive synthesis of the available evidence to clarify patterns and identify consistent outcomes. Tools such as the 6-minute walk test (6MWT), spirometry, and oxygen consumption are crucial for enhancing our understanding of physical and cardiorespiratory impairments in long COVID individuals. They provide tangible, quantifiable insights into the compromised cardiorespiratory function and physical capacities often seen in long COVID patients. The 6MWT is an effective measure of functional exercise capacity, critical for assessing a patient’s ability to perform daily tasks [33,34]. Spirometry is essential for evaluating lung function, identifying potential respiratory impairments common post-COVID-19 [35]. Additionally, monitoring oxygen consumption during physical exertion provides a precise assessment of cardiorespiratory health [36,37]. A better understanding of physical and cardiorespiratory impairments in long COVID is essential for guiding targeted rehabilitation. Identifying consistent patterns will help clinicians develop effective interventions that improve patients’ functional capacity and well-being.

To date, no systematic review has summarized the literature on physical and cardiorespiratory impairments in long COVID. A thorough summary of these impairments would help clinicians and researchers identify key areas for targeted rehabilitation in this growing population. Thus, the purpose of this study was to conduct a systematic review to summarize the physical and cardiorespiratory impairments observed in people with long COVID.

## Methods

This systematic review was conducted according to the Preferred Reporting Items for Systematic Reviews and Meta-Analyses (PRISMA) guidelines [38] and was registered prospectively on PROSPERO (CRD42022352812). There is no published protocol for this systematic review.

### Literature search and study identification

A database search was conducted in MEDLINE, Embase, CINAHL and Web of Science on August 10, 2022, with the assistance of two librarians from *Université Laval* and *Université de Montréal*. The search was updated on February 1, 2024. The search strategy focused on two key areas: the population (adults with long COVID) and the outcomes of interest (physical and cardiorespiratory impairments). The search included a combination of MeSH terms and keywords related to SARS-CoV-2 infection (e.g., “SARS”, “coronavirus”, “COVID*”), long COVID (e.g., “long-term COVID”, “chronic COVID”, “post-acute COVID*”), and physical/cardiorespiratory impairments (e.g., “spirometry”, “6-minute walk test”, “VO2”, “pulmonary function”, “exercise”, “oxygen saturation”). The complete search strategy is available in S1 File. In addition, reference lists of included articles were manually screened to ensure that all relevant studies were included.

### Study selection

Covidence software (Veritas Health Innovation Ltd company, Melbourne, Australia) was used for study selection process. After duplicates removal, titles and abstracts were independently reviewed by at least two of the authors (IS, IZ, MOD). Full-text articles of potentially relevant studies were then obtained and screened to determine eligibility based on inclusion criteria. Preprint studies were excluded to ensure that only peer-reviewed research was included in our analysis. A consensus between two authors was required for article inclusion, with disagreements resolved through discussion with a third reviewer (JSR). The inclusion criteria were: 1) studies involving adults with long COVID (>3 months); 2) at least one outcome related to physical function (Short performance physical battery [SPPB], or sit to stand [STS], six-minute walk test [6MWT]), or any cardiorespiratory and metabolic parameters (oxygen consumption [VO_2_], forced vital capacity [FVC], forced expiratory volume [FEV1], total lung capacity [TLC], diffusing capacity of the lungs for carbon monoxide [DLCO], or arterial oxygen saturation [SpO_2_]); 3) cross-sectional or cohort studies; and 4) written in English or French. Additionally, included studies needed to compare data from long COVID participants with a control group with no history of COVID-19 or present data that could be compared to normative values. Studies were excluded if they were: (1) retrospective studies, case studies, case reports, and reviews, and (2) focused primarily on interventions, rather than assessing physiological or functional impairments.

### Methodological quality assessment

A pair of raters (IS, MOD) independently evaluated the quality of each article that met the inclusion criteria using the Joanna Briggs Institute Critical Appraisal Tools (JBI-CAT) [39]. This risk of bias appraisal tool consists of eight items rated as “Yes”, “No”, “Unclear” or “Not/Applicable”. The items assess: 1) clear definition of inclusion criteria, 2) detailed description of study subjects and settings, 3) validity and reliability of exposure measured, 4) use of objective, standard criteria for measurement of the condition, 5) identification of confounding factors, 6) use of strategies to deal with confounding factors, 7) measurement of the outcomes in a valid and reliable way, and 8) use of appropriate statistical analyses.

The raters first conducted a calibration review by independently evaluating three articles and then discussing each item to clarify the meaning and interpretation of critical appraisal criteria. Then they independently evaluated the remaining included articles. A consensus meeting was held to resolve any disagreements and reach a consensus on the quality ratings for each included study. Pre-consensus inter-rater agreement was evaluated for each item using Cohen’s Kappa coefficient.

### Data extraction

Relevant information regarding the study populations was extracted from the included articles: number of participants, age, sex, body mass index (BMI), percentage of smokers, percentage of hospitalized participants, vaccination status, and time since COVID-19 infection, when applicable. Quantitative data on outcomes was also extracted: SPPB, Distance of the 6MWT, VO_2peak_ and/or VO_2max_, FVC, FEV1, TLC, DLCO, and SpO_2_. A data extraction form was created in Microsoft Excel 2020 (Microsoft, Redmond, United States). Three independent authors (IS, MOD, AR) extracted the data, and then met to reach a consensus.

## Outcomes of interest

### Physical function tests

#### Short Physical Performance Battery (SPPB)*.*

The SPPB test is designed to measure functional status and physical performance using tasks that mimic daily activities [40]. It contains three components: the ability to stand for up to ten seconds with feet positioned in three ways (together side-by-side, semi-tandem and tandem); time needed to complete a 3-meter or 4-meter walk; and time needed to rise from a chair five times. Total score varies between 0 and 12 [41]. Its validity and reliability to assess functional capacity have been confirmed in different adult populations [42–44].

#### Sit to Stand (STS).

The STS was developed to evaluate lower limb function. This test measures the maximum number of sit-to-stand repetitions from a chair that an individual is able to perform during a pre-determined time interval (usually 30 seconds to 1 minute) or the time needed to complete a pre-determined number of repetitions (usually five). Validity, reliability and responsiveness have been shown in different adult populations [45–47].

#### 6-minute Walk Test (6MWT).

The 6MWT test is a versatile test used to assess functional capacity in patients with a wide range of pulmonary, cardiovascular, neurological and neuromuscular disorders [48]. The test consists of walking the longest distance possible in six minutes by going back and forth over a distance of 30 meters (some studies use a 20-meter or 15-meter length). Validity, reliability and responsiveness of this test have been evaluated in different populations [33,34,49].

### Cardio-respiratory and metabolic performance parameters

#### VO_2peak_.

VO_2max_ represents the maximum rate of oxygen consumption by the body during an effort. It is usually measured by tracking oxygen intake during an exercise test. During any effort, VO_2_ increases with incremental intensity, so cardiac output (the product of heart rate and stroke volume), CaO_2_ and CvO_2_ (O_2_ contents of arterial and mixed venous blood, respectively) reach their maximal limits and as a result, a plateau of VO_2_ occurs. This plateau is called VO_2max_. However, many individuals do not reach this plateau due to discomfort or other factors. In such cases, the highest VO_2_ reached, termed VO_2peak_, is used as an estimate of VO_2max_. VO_2max_/VO_2peak_ values can be reported either as an absolute measure in L/min or normalized for body weight, expressed in mL/min/kg [50].

#### Forced Vital Capacity (FVC).

FVC is the total volume of air that can be forcibly exhaled from the lungs after taking the deepest possible breath*.* It measures the overall capacity to expel air and is commonly used to assess lung function and diagnose a range of pulmonary conditions [35,51].

#### Forced Expiratory Volume (FEV_1_).

The FEV_1_ is the volume of air expelled in the first second of a forceful exhalation following a maximal inhalation. It is typically measured in liters [35]. This measurement is obtained during a spirometry test, where the individual takes a deep breath and then exhales as forcefully and rapidly as possible into a mouthpiece connected to a spirometer [35,51].

#### Total Lung Capacity (TLC).

The TLC is the maximum volume of air that the lungs can hold after a maximal inhalation effort [35,51,52]. It is a critical measurement in assessing respiratory function and is used to diagnose and monitor various lung conditions [51].

#### Diffusing Capacity of the Lungs for Carbon Monoxide (DLCO).

DLCO is a pulmonary function test that measures how effectively gases are transferred from the alveoli in the lungs to the blood in the pulmonary capillaries [53]. During the test, the individual inhales a small amount of carbon monoxide along with an inert gas, holds their breath for about 10 seconds, and then exhales [53].

#### Arterial oxygen saturation (SpO_2_).

SpO_2_ is the fraction of oxygen-saturated hemoglobin in relation to total hemoglobin in the blood. The human body requires and regulates a very precise and specific balance of oxygen in the blood. Normal levels of oxygen saturation in arterial blood in humans are 95–99% [54]. It is usually measured with a pulse oximeter, which is a non-invasive device placed over a person’s finger. It measures light wavelengths to determine the ratio of the current levels of oxygenated hemoglobin compared to deoxygenated hemoglobin [55].

#### Data analyses.

Given the heterogeneity in study designs, measurement protocols, control groups, and study populations (e.g., time since infection, hospitalization status, presence of comorbidities), conducting meta-analyses for the different outcomes was not feasible. Therefore, descriptive statistics were used to summarize groups characteristics and outcomes. Results were qualitatively synthesized. Data from studies were compared with age and sex-specific reference values for all outcomes (SPPB, 6MWT, VO_2peak_ and/or VO_2max_, FVC, FEV_1_, TLC, DLCO, and SpO_2_) [35,45,51,53,54,56–63].

## Results

### Literature search and study selection

**Fig 1:**
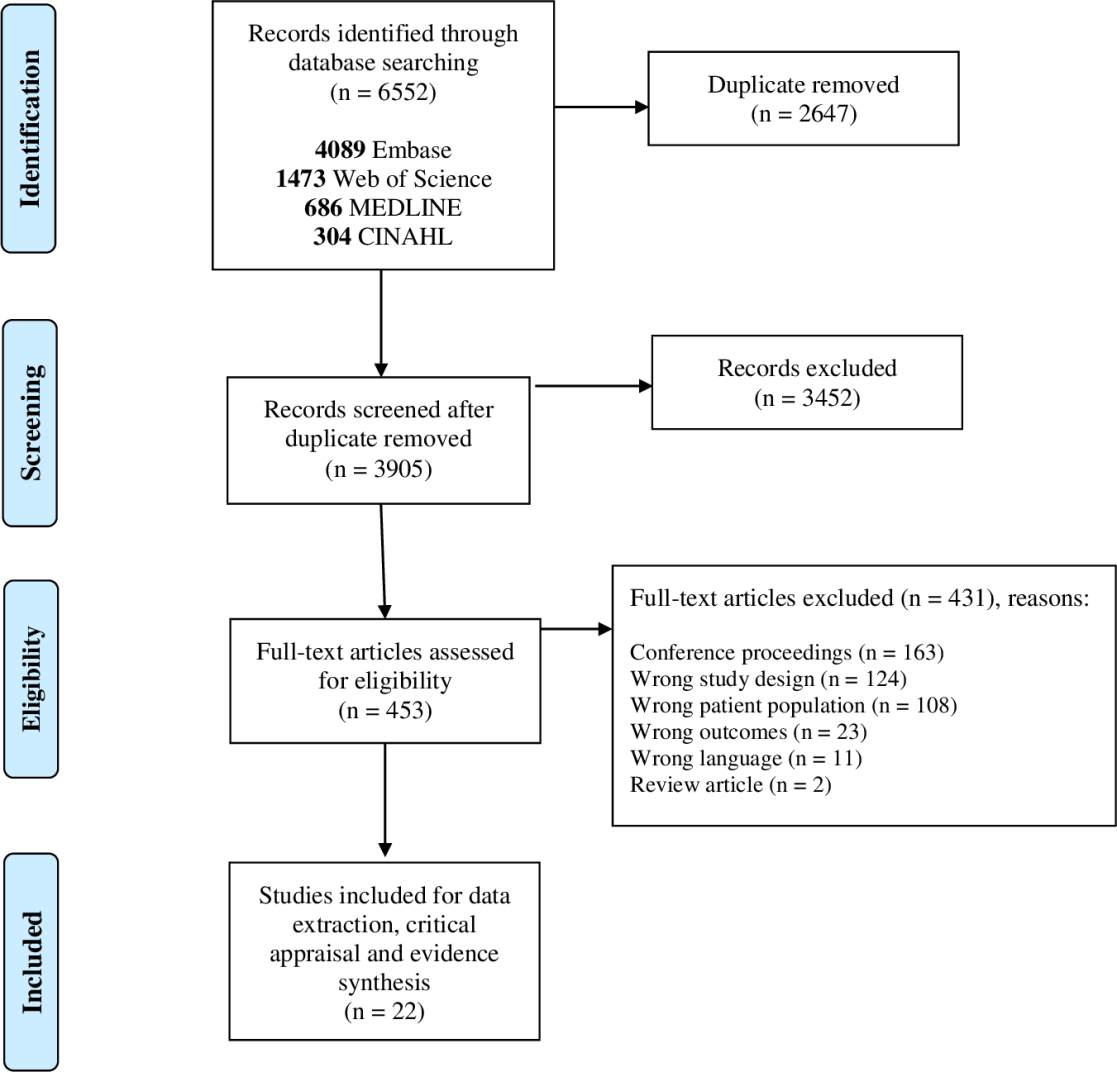
Flowchart of selected studies.

The PRISMA flowchart for study selection is presented in Fig 1. The literature search yielded a total of 6,552 citations. After removing duplicates, titles and abstracts of 3,905 studies were screened, and 453 full-text articles were assessed for eligibility. Of these, 431 studies were excluded, resulting in the inclusion of 22 studies [44,64–84]. These studies involved a total of 124 healthy control adults (with no prior history of COVID-19 infection), 49 individuals with short COVID (a history of COVID-19 infection without persistent symptoms), and 3,041 adults with long COVID.

### Characteristics of included studies

The detailed characteristics of included studies are presented in Table 1. Six studies compared adults with long COVID to a control group [64,65,77,79,83,84], while the remaining 16 studies [66–76,78,80–82,85] either included only one group or compared adults with long COVID to those who had previously been infected but were no longer symptomatic. To assess physical and cardiorespiratory impairments, 11 studies used the 6MWT [64,66,68,69,72,74,77,78,82,84,85], five studies used the STS [68,72,75,80,81], and two studies used the SPPB [68,71]. Cardio-respiratory and metabolic parameters were assessed in 17 studies, using various outcomes including VO_2_, FEV1, FVC, TLC, DLCO, and SpO_2_ [64–70,73–76,78,79,81,83–85].

**Table 1:** Detailed characteristics of included studies.

Study Authors	Study design	Location	Characteristics of Long COVID population								Control group	Outcomes
			n	Gender	Age	BMI	Smoker	Vaccination status	% of hospitalised	Time since COVID infection		
							n (%)					
Aparisi et al. (2021) [78]	Single-center prospective study	Spain	41	11M 30W	54.9 ± 10.5	28 ± 4.9	NR	NR	75.6	181 ± 42 days	No	6MWT VO_2peak_ SpO_2_
Asimakos et al. (2023) [64]	Observationnel, single-center study	Greece	68	30M 38W	56 (46–63)	29.0 (24.9–33.1)	20 (29.4)	NR	73.5	139 (86–350) days	Yes	6MWT FVC FEV1 DLCO
Barisione et al. (2023) [65]	Cross sectional study	Italy	32	25M 7W	56.3 ± 11.2	30 ± 4	16 (50)	NR	26	98-686 days	Yes	FVC FEV1 TLC DLCO SpO_2_
Beaudry et al. (2022) [79]	Cross-sectional study	Canada	28	8M 20W	40 ± 11	24.7 ± 3.1	4 (14.3)	0/28 (0%)	14.3	214 ± 85 days	Yes	VO_2peak_ SpO_2_
Beyer et al. (2023) [66]	Cross-sectional study	Germany	69	23M 46W	46 ± 12	28.5 [11.1]	20 (29.0)	2 Not vaccinated 26 Twice 11 thrice	NR	43 ± 32 weeks	No	6MWT VO_2peak_ FEV1
Colosio et al. (2023) [67]	Retrospective observational study	Italy	11	4M 7W	54 ± 11	23 ± 3	NR	NR	0	8 ± 2 months	Yes Short COVID	VO_2peak_ FVC FEV1 DLCO
doNascimento et al. (2023) [68]	Cross-sectional study	Brazil	135	94M 41W	56.9 ± 13.3	27.9 ± 4.8	2 (1.5)	NR	52.6	1.45 ± 0.7 months after recovery	No	SPPB 6MWT 5STS FVC FEV1
DosSantos et al. (2024) [69]	A cross-sectional study	Brazil	69	36M 33W	53.3 ± 13.2	33.0 ± 5.3	NR	NR	100	3 (2–6) months	No	6MWT FVC FEV1
Evans et al. (2023) [71]	Multicentre prospective, longitudinal cohort study	United Kingdom	1079	NR	NR	31.6(28.0 - 36.4)	NR	NR	NR	median: 157 days IQR: 119–189 days	No	SPPB
Gryglewska et al. (2023) [70]	Not defined	Poland	82	35M 47W	Mean of 54	26.8 (23.2–30.4)	11%	NR	100	NR	No	VO_2peak_ FVC FEV1
Gunnarsson et al. (2023) [72]	Cross sectional study	Denmark	292	128M 164W	51.9 ± 15.2	27.3 ± 12.1	16 (6.4) Missing data: 41	NR	50.3	217.2 ± 111.5 missing n = 102	No	6MWT
Jennings et al. (2022) [80]	Cross-sectional observational study	Ireland	108	32M 76W	46.3 ± 10.3	27.9 ± 4.9	44 (41)	64.8% [fully vaccinated]	21	323.4 ± 184.5 days Range: 111–655	No	5STS
Jimeno-Almazan et al. (2022) [81]	Observational cross-sectional study	Spain	72	25M 47W	45.5 ± 9.0	26.9 ± 4.8	4 (5.6)	40/72 (56% [one dose]]	0	36.3 ± 21.1 weeks	No	5STS VO_2peak_
Kersten, Hoyo et al. (2022) [73]	Cohort study	Germany	120	46M 74W	49.7 ± 15.2	25.4 ± 4.3	24.2% (current/past smoking)	NR	15.8	227 ± 114 days	No	VO_2peak_
Kersten, Wolf et al. (2022) [82]	Cross-sectional study	Germany	367	156M 211W	47.3 ± 14.8	25.8 ± 4.8	68 (18.6)	NR	6.8	179.9 ± 104.5 days	No	6MWT
Kooner et al. (2022) [84]	Multicentre prospective cohort study	Canada	76	38M 38W	53 ± 12	30 ± 5	NR	NR	30.3	Mean: 13.8 ± 8.5 weeks 12.0 (5.0–53.4) weeks	Yes	6MWT SpO_2_
Lacavalerie et al. (2022) [83]	Cross-sectional study	France	33	18M 15F	58 ± 10	34 ± 5	NR	NR	1	197.4 ± 13.1 days	Yes	VO_2peak_ SpO_2_
Niebauer et al. (2023) [74]	Prospective registry	Austria	113	65 M 48 W	56.48 ± 12.56	29.75 ± 4.99	NR	0	NR	6.1 ± 1.7 months.	Yes Hospitalized Without persistent symptoms	6MWT SpO_2_
Njoten et al. (2023) [75]	Cross-sectional study	Norway	65	11 M 54 W	39.0 ± 11.8 39 (19-65)	26.5 ± 5.1	NR	NR	0	9.4 (4.7) months	No	VO_2peak_ FVC FEV1 TLC DLCO SpO_2_
Oliveira et al. (2023) [85]	Cross-sectional study	Brazil	16	2 M 14W	57 (50–59)	32 (30–36)	0	NR	0	98 (93–106) days	No	6MWT VO_2peak_ FVC FEV1 SpO_2_
Philippe et al. (2023) [76]	Prospective monocentric cohort study	France	137	93 M 44 F	55 (46.5–66.5).	25.5 (23.0–28.4)	9 (6.6)	NR	61.3	202 (105–611) days.	No	FVC DLCO SpO_2_
Yu et al. (2022) [77]	Prospective study	Sweden	28	7M 21W	46.5 ± 8	26 ± 5.1	0%	NR	0	7.7 ± 3.6 months	Yes	6MWT

LCG long COVID group, BMI body mass index, 6MWT six-minute walk test, VO_2peak_ maximal oxygen consumption, STS sit to stand, SPPB Short physical performance battery, SpO_2_ arterial oxygen saturation, FEV1 forced expiratory volume, FVC forced vital capacity, TLC total lung capacity, DLCO diffusing capacity of the lungs for carbon monoxide, NA not applicable, NR not reported.

### Risk of bias of included studies

**Fig 2:**
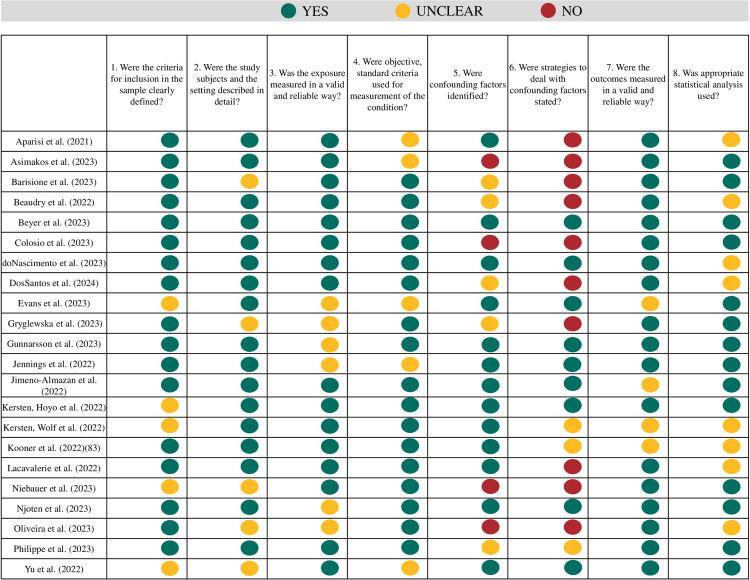
Risk of bias assessment of included studies.

The risk of bias assessment, as evaluated by the JBI-APT, is presented in Fig 2. The overall strengths of the studies included a clear definition of inclusion criteria (Item 1; 17/22 “yes”), a detailed description of study subjects and settings (Item 2; 17/22 “yes”), and valid and reliable measurement of exposure (Item 3; 16/22 “yes”). The main weaknesses were a lack of identification of confounding factors (Item 5; 4/22 “no” and 5/22 “unclear”) and insufficient strategies to address these confounding factors (Item 6; 10/22 “no”). Pre-consensus inter-rater agreement across items ranged from moderate to perfect, with Cohen’s Kappa values ranging from 0.4 to 1.0.

### Outcome results

#### Short Physical Performance Battery (SPPB).

DoNascimento et al. (2023) reported a mean (SD) SPPB score of 11.7 ± 0.9 in 135 adults with long COVID [68]. In contrast, Evans et al. (2022), found that 58.8% of their sample of 1,079 long COVID adults had a mean SPPB score below 10 [71]. Notably, Evans et al’s study reported a higher average BMI among participants (31.6 [28.0–36.4]) compared to Do Nascimento et al’s study (27.9 ± 4.8), which may account for the difference. When compared to reference values (score> 11.5) [59], adults with long COVID had a lower mean SPPB score only in Evans et al’s study. However, the findings from just two studies are insufficient to draw definitive conclusions.

#### Sit-to-Stand Test (STS).

Three studies [68,80,81] used the five-repetition STS (5STS) test. Only one study [80] reported lower performance on the 5STS (mean time: 14.3 ± 9.2 s) in a sample of 108 long COVID adults when compared to reference values (mean time between 7.5 and 8.6s) [59]. Meanwhile, the study by Do Nascimento et al. reported a mean time of 8.7 ± 4.5 seconds in 135 adults aged 56.9 ± 13.3 years [68], and the Jimeno-Almazan study noted a mean time of 7.6 ± 2.8 seconds among 72 adults averaging 45.5 ± 9.0 years [81].

#### Six-minute Walk test (6MWT).

Data were considered normal if they were at or above the 50^th^ percentile of normative data. All eleven studies that used the 6MWT reported reduced walking distance for adults with long COVID compared to reference values [58]. For instance, Gunnarsson et al. (2023) observed a mean distance of 489.5 ± 138.7 meters in a sample of 292 long COVID adults (mean age: 51.9 ± 15.2 years), while a minimal distance of 585 meters is expected for healthy adults aged 50–59 years [58].

Only two studies compared their results to control or asymptomatic groups. Niebauer et al. (2023) found no significant difference between long COVID patients (aged 56.5 ± 12.6, 6MWT: 549.6 ± 97.3 meters) and an asymptomatic group (age: 53. 7 ± 12.8, 6MWT: 577.7 ± 104.1 meters), though the asymptomatic group has a higher mean BMI (30.16 (4.80)), which might explain similarity in result. In contrast, Yu et al. (2022) reported a significant difference (p = 0.001) between the control group (age 44.1 ± 10.8, 6MWT: 678 ± 78 meters) and the long COVID group (age 46.5 ± 8, 6MWT: 583 ± 111 meters), with both groups having a mean BMI around 25. Detailed results are presented in Table 2.

**Table 2:** 6MWT results.

Study ID	Gender/Sex		Age		BMI		n (%) of smokers		6MWT (m)	
	Control	LCG	Control	LCG	Control	LCG	Control	LCG	Control	LCG
Aparisi et al. (2021)	NA	11M 30W	NA	54.9 ± 10.5	NA	28 ± 4.9	NA	NR	NA	535 (467–600)
Asimakos et al. (2023)	NA	30M 38W	NA	56 (46–63)	NA	29.0 (24.9–33.1)	NA	20 (29.4)	NA	474 (378–558)
Beyer et al. (2023)	NA	23M 46W	NA	46 ± 12	NA	28.5 ± 11.1	NA	20 (29.0)	NA	525 ± 88 *W: 516 ± 83* *M: 543 ± 96*
doNascimento et al. (2023)	NA	94M 41W	NA	56.9 ± 13.3	NA	27.9 ± 4.8	NA	2 (1.5)	NA	517.7 ± 103.3 *hospitalized (n = 71): 502 ± 113.2* *non-hospitalized (n = 64): 538.4 ± 86.8*
DosSantos et al. (2024)	NA	36M 33W	NA	53.3 ± 13.2	NA	33.0 ± 5.3	NA	NR	NA	72.3 ± 15.5% of predicted value
Gunnarsson et al. (2023)	NA	128M 164W	NA	51.9 ± 15.2	NA	27.3 ± 12.1	NA	16 (6.4) Missing data: 41	NA	489.5 ± 138.7 *non-hospitalized: n = 99,* *507.9 ± 121.5* *hospitalized: n = 81,* *467.0 ± 155.0*
Kersten, Wolf et al. (2022)	NA	156M 211W	NA	47.3 ± 14.8	NA	25.8 ± 4.8	NA	68 (18.6)	NA	523.5 ± 77.7
Kooner et al. (2022)	NA	38M 38W	NA	53 ± 12	NA	30 ± 5	NA	NR	NA	454 ± 84
Niebauer et al. (2023)	Asymptomatic patients 25M 12W	65 M 48 W	53.67 ± 12.80	56.5 ± 12.6	30.2 ± 4.8	29.75 ± 4.99	NR	NR	Asymptomatic patients 577.7 ± 104.10	549.6 ± 97.30
Oliveira et al. (2023)	NA	2 M 14W	NA	57 (50–59)	NA	32 (30–36)	NA	0 (0)	NA	434 (386–478)
Yu et al. (2022)	6M 16W	7M 21W	44.1 ± 10.8	46.5 ± 8	25 ± 3.4	26 ± 5.1	0	0 (0)	678 ± 78	583 ± 111

LCG long COVID group, BMI body mass index, 6MWT six-minute walk test, NA not applicable, NR not reported.

#### Oxygen consumption (VO_2peak_).

Three studies [75,79,81] found no significant decrease in VO_2peak_ values in adults with long COVID when compared to reference values [56], with only one of these studies comparing results to a control group [79]. This study reported lower VO_2peak_ values in the long COVID group (32 ± 9.0 ml/kg/min) compared to the control group (40 ± 9.0 ml/kg/min), though both values remained within the normal range [56]. However, seven other studies [66,67,70,73,78,83,85] reported a reduced VO_2peak_ when compared to reference values [56]. For instance, Colosio et al. (2023) reported a reduced VO_2peak_ of 24.7 (5.0) ml/min/kg in the long COVID group, while the control group had a VO_2peak_ of 32.9 (7.4) ml/min/kg. One study [83] reported a reduced VO_2peak_ (15.7 ± 5.0 mlO_2_/min/kg) in both the long COVID and control groups compared to reference values (28–35 mlO_2_/min/kg) [56]. However, participants in this study, both with and without long COVID, were obese, and no significant difference in VO_2peak_ was identified between the two groups. The reduced VO_2peak_ value observed may be attributable to the high BMI (34.0 ± 5.0 and 41.0 ± 8.0, respectively) and other related health conditions in this sample [86]. Detailed results are presented in Table 3.

**Table 3:** VO_2peak_ results.

Study ID	Gender/Sex		Age		BMI		n (%) of smokers		VO_2peak_	
	Control	LCG	Control	LCG	Control	LCG	Control	LCG	Control	LCG
Aparisi et al. (2021)	NA	11M 30W	NA	54.9 ± 10.5	NA	28 ± 4.9	NA	NR	NA	17.8 (15.8–21.2)
Beaudry et al. (2023)	7M 17W	8M 20W	41 ± 12	40 ± 11	23.6 ± 3.2	24.7 ± 3.1	3 (12.5)	4 (14.3)	40 ± 9	32 ± 9
Beyer et al. (2023)	NA	23M 46W	NA	46 ± 12	NA	28.5 ± 11.1	NA	20 (29.0)	NA	22.5 ± 6.4
Colosio et al. (2023)	Asymptomatic patients: 6M 6W	4M 7W	49 ± 9	54 ± 11	24 ± 2	23 ± 3	NR	NR	Asymptomatic patients: 32.9 ± 7.4	24.7 ± 5.0
Gryglewska et al. (2023)	NA	35 M 47 F	NA	average age of 54	NA	26.79 (23.24–30.42)	NA	9 (11)	NA	21.00 (17.00–26.00)
Jimeno-Almazan et al. (2022)	NA	25M 47W	NA	45.5 ± 9.0	NA	26.9 ± 4.8	NA	4 (5.6)	NA	35.8 ± 10.4
Kersten, Hoyo et al. (2022)	NA	46 M 74 W	NA	49.7 ± 15.2	NA	25.4 ± 4.3	NA	29 (24.2)	NA	24.6 ± 7.1
Lacavalerie et al. (2022)	6M 23W	18M 15W	50 ± 13	58 ± 10	41 ± 8	34 ± 5	NR	NR	15.3 ± 2.7	15.7 ± 5.0
Njoten et al. (2023)	NA	11 M 54 W	NA	39.0 ± 11.8 39 (19-65)	NA	26.5 ± 5.1	NA	NR	NA	31.1 (6.4)
Oliveira et al. (2023)	NA	2 M 14W	NA	57 (50–59)	NA	32 (30–36)	NA	0	NA	19 (14–37)

LCG long COVID group, BMI body mass index, VO_2_ oxygen consumption, NA not applicable, NR not reported.

#### FVC, FEV1, TLC.

FVC, FEV1, and TLC were evaluated in 10 studies [64–70,75,76,85]. All studies reported normal values when compared to reference values [35,51]. For example, FVC and FEV1 were consistently above the predicted lower limit in adults with long COVID, indicating preserved lung function. Similarly, TLC values remained within expected norms, suggesting no significant restrictive lung impairment. Detailed results are presented in Table 4.

**Table 4:** Spirometry results.

Study ID	Gender/Sex		Age		BMI		n (%) of smokers		DLCO		Spirometry	
	Control	LCG	Control	LCG	Control	LCG	Control	LCG	Control	LCG	Control	LCG
Aparisi et al. (2021)	NA	11M 30W	NA	54.9 ± 10.5	NA	28 ± 4.9	NA	NR	NA	NA	NA	Resting: 97 (96–98) Peak: 97 (96–98)
Asimakos et al. (2023)	NA	30M 38W	NA	56 (46–63)	NA	29.0 (24.9–33.1)	NA	20 (29.4)	NA	% pred: 67 ± 18.6%	NA	FEV1 (% pred): 94.3 (81.6–105.0) FVC (% pred): 93.8 (80.1–102.7)
Barisione et al. (2023)	1 W 19 M	7 W 25 M	50.4 ± 9.8	56.3 ± 11.2	26 ± 3	30 ± 4	10 (50)	16 (50)	30.8 ± 3.82 % pred 110 ± 13	22.5 ± 4.58 mL/min/mmHg % pred: 89 ± 16	SpO_2_: 97.6 ± 0.7% FVC: 4.96 ± 0.69 FEV: 3.95 ± 0.46 TLC: 7.00 ± 0.93	SpO_2_: 97.3 ± 0.9 FVC:4.06 ± 0.79 FEV: 3.29 ± 0.62 TLC: 5.63 ± 1.04
Beaudry et al. (2023)	7M 17W	8M 20W	41 ± 12	40 ± 11	23.6 ± 3.2	24.7 ± 3.1	3 (12.5)	4 (14.3)	NA	NA	SpO_2_: 96 ± 3%	SpO_2_: 96 ± 3%
Beyer et al. (2023)	NA	23M 46W	NA	46 ± 12	NA	28.5 ± 11.1	NA	20 (29.0)	NA	NA	NA	FEV1 (ml): 3055 [970]
Colosio et al. (2023)	Asymptomatic patients: 6M 6W	4M 7W	49 ± 9	54 ± 11	24 ± 2	23 ± 3	NR	NR	DLCO/VA 106 ± 11	DLCO/VA 98 ± 12%	FVC: 114 ± 23% of predicted value FEV1: 112 ± 26% of predicted value	FVC (% pred): 117 ± 15 FEV1 (% pred): 119 ± 17%
doNascimento et al. (2023)	NA	94M 41W	NA	56.9 ± 13.3	NA	27.9 ± 4.8	NA	2 (1.5)	NA	% pred: 74 ± 17.5 HP (n = 71): 69.0 ± 16.5 NHP (n = 64): 80 ± 17.4	NA	FEV1 (L) 2.8 ± 0.8 FEV1 (% pred): 82.7 ± 13.9
DosSantos et al. (2024)	NA	36M 33W	NA	53.3 ± 13.2	NA	33.0 ± 5.3	NA	NR	NA	NA	NA	FEV1, (% pred): 85.3 ± 15.1 FVC, (% pred): 86.5 ± 13.9 FEV1/FVC, (% pred): 85.1 ± 15.2
Gryglewska et al. (2023)	NA	35 M 47 F	NA	Mean age of 54	NA	26.79 (23.24–30.42)	NA	9 (11)	NA	NA	NA	FEV1 (L): 2.99 (2.55–3.56) FVC: 3.79 (3.18–4.44)
Kooner et al. (2022)	NA	38M 38W	NA	53 ± 12	NA	30 ± 5	NA	NR	NA	NA	SpO_2_ rest: 97 ± 2	SpO2 rest: 97 ± 2 SpO_2_ post exertion: 97 ± 3
Lacavalerie et al. (2022)	6M 23W	18M 15W	50 ± 13	58 ± 10	41 ± 8	34 ± 5	NR	NR	NA	NA	SpO_2_: 98 ± 2	SpO_2_: 96 ± 3
Niebauer et al. (2023)	Asymptomatic patients 25M 12W	65 M 48 W	53.7 ± 12.8	56.5 ± 12.6	30.2 ± 4.8	29.75 ± 4.99	NR	NR	NA	NA	asymptomatic patients SpO_2_: 98.25 ± 1.15	SpO_2_: 97.62 ± 1.29
Njoten et al. (2023)	NA	11 M 54 W	NA	39.0 ± 11.8 39 (19-65)	NA	26.5 ± 5.1	NA	NR	NA	% pred: 87.4 (11.3)	NA	SpO_2_ Rest: 99 (1) SpO_2peak_: 96 (3) FEV1 (% pred) 96.6 (10.7) FEV1 (L): 3.3 (0.6) FVC (L): 4.2 (0.7) FVC (% pred) 100.8 (10.9) TLC (% pred) 97.7 (11.4)
Oliveira et al. (2023)	NA	2 M 14W	NA	57 (50–59)	NA	32 (30–36)	NA	0	NA	NA	NA	Basal SO2 (%) 96 (93–98) End of test SO2 (%) 94 (92–96) FVC (% pred): 93 (88–103) FEV1 (% pred) 96 (88–102) FEV1/FVC (%) 84 (76–89) FEF25 − 75% (% pred) 114 (74–126)
Philippe et al. (2023)	NA	93 M 44 W	NA	55 (46.5–66.5)	NA	25.5 [23.0–28.4]	NA	9 (6.6)	NA	% pred (Median [IQR]) 73.0 [61.0–83.0]	NA	SaO2 (%): 96.0 [95.0–98.0] FVC- (% pred): 95.0 [80.0–107.5]

LCG long COVID group, BMI body mass index, SpO_2_ arterial oxygen saturation, FEV1 forced expiratory volume, FVC forced vital capacity, TLC total lung capacity, DLCO diffusing capacity of the lungs for carbon monoxide, DLCO/VA lung diffusing capacity normalized to the alveolar volume, NA not applicable, NR not reported.

#### Diffusing Capacity of the Lungs for Carbon Monoxide (DLCO).

Six studies reported DLCO in adults on long COVID [64,65,67,68,75,76], with one study including a control group of healthy adults [65], and another comparing individuals with long COVID to those without persistent symptoms [67]. The study with a control group found a significant difference in DLCO between the control group (30.8 ± 3.8 mL/min/mmHg) and adults with long COVID (22.5 ± 4.6 mL/min/mmHg, p < 0.001) [65]. However, their values remained within the normal range (89 ± 16% of predicted values). In contrast, the second study reported no significant difference in DLCO between asymptomatic individuals and those with long COVID, with values exceeding 95% of predicted values [67]. Among the remaining studies, three reported abnormal DLCO values with predicted values below 75% [64,68,76] while the remaining study found normal values with exceeding 80% of predicted values [75]. Detailed results are presented in Table 4.

#### Arterial oxygen saturation (SpO_2_).

All nine studies [65,74–76,78,79,83–85] that reported SpO_2_ values in adults with long COVID showed no reduction in SpO_2_ compared to reference values [54]. Five of these studies included a control group [65,74,79,83,84]. Only one study [74] found a significant difference in SpO_2_ between long COVID adults (97.6 ± 1.3) and asymptomatic individuals (98.3 ± 1.2, p = 0.03), though both values were within normal range [54]. Detailed results are presented in Table 4.

## Discussion

The objective of this systematic review was to summarize the physical and cardiorespiratory impairments observed in individuals with long COVID. Our findings highlight a complex and nuanced impact of long COVID on pulmonary function and exercise capacity. While parameters such as FVC, FEV1, TLC, and SpO_2_ generally remain within normal ranges, indicating preserved lung volumes and capacities, notable reductions in 6MWT, DLCO and VO_2peak_ suggest a significant decline in exercise capacity.

When examining 6MWT results, adults with long COVID appear to have diminished physical capacity. All eleven studies reported a reduced walking distance in the 6MWT, with participants walking shorter distances than their age-adjusted predicted values [58]. This reduced capacity is likely due to extended periods of illness and inactivity during and after COVID-19 infection, leading to reduced cardiovascular and respiratory fitness. This is further supported by our VO_2peak_ findings, where adults with long COVID exhibited lower VO_2peak_ values. For instance, Beyer et al’s study showed that long COVID participants reached only 72.3 ± 18.5% of their predicted VO_2peak_, which correlates with lower 6MWT distance according to sex and age [66]. Longitudinal studies, though limited, provide key insights into the persistence and potential recovery of physical capacity in long COVID patients. A prospective study by O’Brien et al. (2022) tracked hospitalized COVID-19 survivors over a year, showing a significant increase in 6MWT distance from 365 ± 209 m at 10 weeks to 447 ± 85 m at one-year post-discharge (F = 10.3, p < 0.001) [87]. However, despite this progress, distances remained below population norms. Similarly, another study reported a significant increase in 6MWT from 459.8m to 499.8m over 6 months, indicating partial recovery but still below age-adjusted norms [88].

Several recent studies have demonstrated that exercise training and breathing exercises can enhance physical capacity in long COVID patients [89–92]. Exercise-based interventions, particularly structured aerobic and resistance training, have been shown to enhance fatigue and physical performance [91]. In parallel, respiratory muscle training has also emerged as a beneficial approach. A randomized controlled trial showed that combining home-based breathing exercises with cardiac rehabilitation significantly improved cardiorespiratory fitness, notably through enhanced 6MWT performance [92]. Furthermore, a systematic review focusing on older adults with long COVID showed that rehabilitation interventions significantly improved 6MWT performance, reduced fatigue, and enhanced independence. Exercise training was particularly effective for physical capacity, while respiratory rehabilitation including diaphragmatic breathing, respiratory muscle training, cough exercises, and thoracic stretching was particularly effective in improving pulmonary function [89]. However, these studies consistently emphasize that long-term, individualized rehabilitation programs are necessary to achieve functional levels comparable to normative data.

These findings highlight the prolonged impact of COVID-19 on physical function and the potential barriers to full recovery. Similar impairment have been observed in other populations, including individuals with chronic obstructive pulmonary disease, pulmonary fibrosis, and myalgic encephalomyelitis/chronic fatigue syndrome (ME/CFS) [93–95]. For example, adults with ME/CFS experience physical impairments exacerbated by post-exertional malaise, a condition driven by autonomic dysfunction and impaired energy metabolism [96,97]. Autonomic dysregulation has also been identified in individuals with long COVID, characterized by heightened sympathetic activity and reduced parasympathetic tone, which significantly contributes to decreased physical capacity [98]. Future studies should investigate these mechanisms more systematically to determine their relative contributions to functional impairment and identify key predictors of long-term recovery.

BMI is often correlated with walking distance [99]. Notably, the mean BMI in adults from the studies assessing 6MWT ranged between 25 and 33, encompassing the categories of overweight (BMI less than 30) and obesity (BMI greater than 30) [100]. A higher BMI is associated with an increase likelihood of functional limitations and decline [101]. Moreover, obesity has been identified as a strong risk factors for the development of long COVID [102]. Evidence also suggests that hormone and nutrient dysregulation in individuals with obesity can alter the response to infection [103], as obesity is linked to several underlying risk factors for COVID-19, including hypertension, dyslipidemia, type 2 diabetes and chronic kidney or liver disease [86].

Our results also suggest reduced DLCO despite normal SpO_2_, FEV1, FVC, and TLC. A low DLCO can independently predict oxygen desaturation during exertion, such as the 6MWT [104]. Even when resting SpO_2_ is normal, physical exertion may reveal impairments in gas exchange efficiency, leading to reduced exercise capacity, as indicated by lower VO_2peak_ and 6MWT performance [105]. This discrepancy suggests that while basic lung mechanics and resting oxygen levels are preserved, adults with long COVID experience significant limitations in sustained physical activities due to compromised gas exchange and deconditioning. Moreover, DLCO is closely tied to pulmonary vasculature and cardiac function. In chronic heart failure, DLCO may be reduced due to changes in the alveolar-capillary membrane and decreased pulmonary blood flow [106]. A lower DLCO in this population is associated with impaired exercise performance, as effective oxygen transfer oxygen from the alveoli to the bloodstream is critical during physical exertion [106]. VO_2peak_ is also influenced by both cardiac output and muscle oxygen extraction [107]. Impairments in cardiovascular function or muscle metabolism can lead to lower VO_2peak_, even when lung function parameters such as SpO_2_, FEV1, and TLC remain within normal ranges [108,109]. For example, a prospective cross-sectional study fund that 69% of hospitalized COVID patients (n = 60) experienced reduced physical function, while only 10% showed a decline SpO_2_ [110].

While variability exists across studies due to differences in study design, patient populations, pre-existing comorbidities, and potential confounding factors such as ethnicity, baseline health status, severity of acute infection, and disparities in post-COVID rehabilitation, our systematic review identifies consistent patterns across the available evidence. The overall trend of reduced exercise capacity is clear. However, future research should address the impact of these confounding factors by using standardized assessment protocols, and matched cohort designs to more accurately distinguish the direct physiological effects of long COVID from external influences.

The key finding of our systematic review is that normal spirometry values (FVC, FEV1, TLC, and SpO_2_) suggest that basic lung function remains within normal values in adults with long COVID. However, these tests do not assess the performance of the lungs and cardiovascular system under stress. The 6MWT and VO_2peak_ may provide more sensitive indicators of exercise capacity limitations that might not be evident at rest. Despite impaired gas exchange efficiency (as reflected by lower DLCO), the respiratory system compensates effectively at rest to maintain adequate blood oxygen levels. This compensation may involve mechanisms such as increased ventilation or enhanced perfusion of well-functioning alveoli.

Despite efforts to synthesize high-quality evidence, this review is subject to residual biases. The included studies vary in sample size, recruitment strategies, and participants comorbidities, potentially introducing selection and reporting biases. The heterogeneity in characteristics such as socioeconomic status, vaccination status, and access to rehabilitation may have influenced the outcomes but were inconsistently reported or controlled for. Additional confounding may stem from unmeasured factors like mental health, medication use, or autonomic dysfunction, which are known to affect post-COVID exercise tolerance and recovery. Although consistent trends in reduced physical capacity were identified, these findings should be interpreted with caution and may not be generalizable to all individuals with long COVID.

## Conclusion

This systematic review highlights the complex and multifaceted nature of physical and cardiorespiratory impairments in individuals with long COVID. While basic pulmonary function parameters often remain within normal ranges, significant reductions in exercise capacity, as indicated by decreased 6MWT distances and VO_2peak_ values, point to substantial challenges in physical function. These findings underscore the need for comprehensive assessments and individualized rehabilitation programs that address cardiovascular fitness, muscle strength, and weight management. Understanding the nuanced impacts of long COVID is crucial for developing effective interventions and improving the quality of life for affected individuals. Standardized assessment protocols and equitable access to multidisciplinary, long-term rehabilitation services are crucial for optimizing patient outcomes. Further research, including larger studies with better control of confounding factors, is warranted to elucidate the underlying mechanisms responsible for these impairments and to refine intervention strategies.

### Clinical implications

The findings of this systematic review highlight several important clinical implications for managing patients with long COVID. Although basic pulmonary function parameters (FVC, FEV1, TLC, and SpO_2_) often remain within normal ranges, more sensitive tests such as the 6MWT and VO_2peak_ should be included in routine assessments to detect subtle exercise capacity limitations. These tests can help identify patients who may benefit from targeted interventions. Additionally, gas exchange efficiency must be evaluated, as reduced DLCO suggests potential exercise-related impairments, even if resting SpO_2_ levels are normal. A multidisciplinary approach is essential to optimize health outcomes. Individualized rehabilitation programs should prioritize improving cardiovascular fitness and muscle strength. From a policy perspective, standardized assessment protocols should be integrated into guidelines to ensure early detection and management of long COVID impairments. Healthcare systems should prioritize access to long-term rehabilitation programs and invest in research to support multidisciplinary care. Policies must also ensure equitable access to these services, especially for vulnerable populations.

### Strengths and limitations

This systematic review followed a rigorous methodological approach, adhering to PRISMA guidelines and employing an extensive search strategy. The inclusion of reliable and responsive tests, such as the 6MWT and VO_2peak_, strengthens the validity of our findings regarding exercise capacity impairments. To enhance understanding and facilitate comparison across studies, we have also summarized the included studies by outcomes.

However, several limitations must be noted. First, most of the included studies did not adequately address confounding factors such as comorbidities, BMI, age, which may impact results. Long COVID has been shown to be more prevalent in populations with pre-existing conditions, so these factors must be considered when interpreting the findings. Future research should aim to adequately control for such confounders. Second, the variability in symptom duration among long COVID participants regarding the time since infection, may impact results. To ensure consistency, we included only studies that explicitly diagnosed long COVID as symptoms persisting for more than three months. When not specified, we used time since infection as a criterion, excluding studies with assessments conducted less than three months post infections. Third, the most severely affected individuals, who may be unable to perform tests like the 6MWT, are likely underrepresented in the studies included, potentially underestimating the true burden of long COVID on physical and cardiorespiratory function. Finaly, while the included studies are diverse in design and outcomes measured, no formal heterogeneity analysis (e.g., I^2^ statistic) was conducted, as this review does not include a meta-analysis. However, we acknowledge this variability and have reported results by outcomes to allow for a clearer interpretation across studies.

## Supporting information

S1 File Complete search strategy. (DOCX)
