# Supplementary material for: The impact of long COVID on physical and cardiorespiratory parameters: A systematic review
Source: PLoS One. 2025 Jun 4;20(6):e0318707. doi: 10.1371/journal.pone.0318707 (PMC12136454; doi:10.1371/journal.pone.0318707)
Supplement: S1 File — (DOCX) [file pone.0318707.s001.docx]

**Medline search strategy:** August 10, 2022

| 1 | (((covid or corona*) adj1 (long-term or chronic or post-acute or long)) or post-covid or long-covid).ti,ab,kf. | 9148 |
| --- | --- | --- |
| 2 | exp Physical Fitness/ or exp spirometry/ or exp Exercise Test/ or exp Walk Test/ or exp Exercise Tolerance/ or exp Physical Exertion/ or exp Respiratory Function Tests/ or exp physical endurance/ | 341133 |
| 3 | ("physical fitness" or "physical endurance" or "aerobic endurance" or "physical functional performance" or "physical function" or "cardiorespiratory fitness" or "physical exertion" or "exercise tolerance" or "exercise test" or ((cardiorespiratory or cardiopulmonary or pulmonary or lung or respiratory) adj1 function) or spiromet* or "diffusing capacity for carbon monoxide" or DLCO or vo2max or "maxim* oxygen consumption" or "oxygen consumption" or "maxim* oxygen uptake" or "peak VO2" or VO2* or "6-minute walk*" or "6 minute walk*" or 6MW* or "walk test" or "stress test " or borg* or SatO2).ti,ab,kf. | 235701 |
| 4 | 2 OR 3 | 472352 |
| 5 | 1 AND 4 | 575 |

**Medline search strategy:** February 1, 2024

| 1 | (((covid or corona*) adj1 (long-term or chronic or post-acute or long)) or post-covid or long-covid).ti,ab,kf. | 17108 |
| --- | --- | --- |
| 2 | exp Physical Fitness/ or exp spirometry/ or exp Exercise Test/ or exp Walk Test/ or exp Exercise Tolerance/ or exp Physical Exertion/ or exp Respiratory Function Tests/ or exp physical endurance/ | 347926 |
| 3 | ("physical fitness" or "physical endurance" or "aerobic endurance" or "physical functional performance" or "physical function" or "cardiorespiratory fitness" or "physical exertion" or "exercise tolerance" or "exercise test" or ((cardiorespiratory or cardiopulmonary or pulmonary or lung or respiratory) adj1 function) or spiromet* or "diffusing capacity for carbon monoxide" or DLCO or vo2max or "maxim* oxygen consumption" or "oxygen consumption" or "maxim* oxygen uptake" or "peak VO2" or VO2* or "6-minute walk*" or "6 minute walk*" or 6MW* or "walk test" or "stress test " or borg* or SatO2).ti,ab,kf. | 256054 |
| 4 | 2 OR 3 | 495669 |
| 5 | 1 AND 4 | 1067 |

**Web of science search strategy:** August 10, 2022

| 1 | sars* OR coronavirus* OR "corona virus*" OR covid* (Topic) | 322,663 |
| --- | --- | --- |
| 2 | "long-term covid*" OR "long covid*" OR "chronic covid*" OR "post-acute covid*" OR post-covid* OR "post-acute corona*" (Topic) | 6319 |
| 3 | spiromet* OR 6mw* OR "6 minute walk*" OR vo2* OR "physical function" OR "pulmonary function" OR "lung function" OR exercise OR borg* OR SatO2 (Topic) | 687,25 |
| 4 | 1 AND 2 AND 3 | 380 |

**Web of science search strategy:** February 1, 2024

| 1 | sars* OR coronavirus* OR "corona virus*" OR covid* (Topic) | 559,38 |
| --- | --- | --- |
| 2 | "long-term covid*" OR "long covid*" OR "chronic covid*" OR "post-acute covid*" OR post-covid* OR "post-acute corona*" (Topic) | 17,837 |
| 3 | spiromet* OR 6mw* OR "6 minute walk*" OR vo2* OR "physical function" OR "pulmonary function" OR "lung function" OR exercise OR borg* OR SatO2 (Topic) | 774,626 |
| 4 | 1 AND 2 AND 3 | 1,24 |

**Cinahl Search strategy:** August 10, 2022

| 1 | (MH "Post-Acute COVID-19 Syndrome") | 306 |
| --- | --- | --- |
| 2 | TI ( ("long-term covid*" OR "long covid*" OR "long-covid" OR"chronic covid*" OR "post-acute covid*" OR post-covid* OR "post-acute corona*") OR AB ( ("long-term covid*" OR "long covid*"OR "long-covid" OR "chronic covid*" OR "post-acute covid*" OR post-covid* OR "post-acute corona*") ) OR MW ( ("long-term covid*" OR "long covid*" OR "long-covid" OR "chronic covid*" OR "post-acute covid*" OR post-covid* OR "post-acute corona*") ) | 1,383 |
| 3 | 1 OR 2 | 1,383 |
| 4 | (MH "Physical Fitness+") OR (MH "Spirometry") OR (MH "Exercise Test+") OR (MH "Exercise Test, Cardiopulmonary") OR (MH "Walking") OR (MH "Respiratory Function Tests") OR (MH "Oxygen Saturation") OR (MH "Oxygen Consumption") OR (MH "Lung Volume Measurements") OR (MH "Spirometry") OR (MH "Respiratory Airflow") | 89,749 |
| 5 | TI ("physical fitness" OR "physical endurance" OR "aerobic endurance" OR "physical functional performance" OR "physical function" or "cardiorespiratory fitness" OR "physical exertion" OR exercise OR "cardiorespiratory function" OR "cardiopulmonary function" OR "pulmonary function" OR "lung function" OR "respiratory function" OR spiromet* OR "diffusing capacity for carbon monoxide" OR DLCO OR vo2max OR "maxim* oxygen consumption" OR "oxygen consumption" OR "maxim* oxygen uptake" OR "peak VO2" OR VO2* OR "6-minute walk*" OR "6 minute walk*" OR 6MW* OR "walk test" OR "stress test " OR borg* OR SatO2) OR AB ("physical fitness" OR "physical endurance" OR "aerobic endurance" OR "physical functional performance" OR "physical function" or "cardiorespiratory fitness" OR "physical exertion" OR exercise OR "cardiorespiratory function" OR "cardiopulmonary function" OR "pulmonary function" OR "lung function" OR "respiratory function" OR spiromet* OR "diffusing capacity for carbon monoxide" OR DLCO OR vo2max OR "maxim* oxygen consumption" OR "oxygen consumption" OR "maxim* oxygen uptake" OR "peak VO2" OR VO2* OR "6-minute walk*" OR "6 minute walk*" OR 6MW* OR "walk test" OR "stress test " OR borg* OR SatO2) OR MW ("physical fitness" OR "physical endurance" OR "aerobic endurance" OR "physical functional performance" OR "physical function" or "cardiorespiratory fitness" OR "physical exertion" OR exercise OR "cardiorespiratory function" OR "cardiopulmonary function" OR "pulmonary function" OR "lung function" OR "respiratory function" OR spiromet* OR "diffusing capacity for carbon monoxide" OR DLCO OR vo2max OR "maxim* oxygen consumption" OR "oxygen consumption" OR "maxim* oxygen uptake" OR "peak VO2" OR VO2* OR "6-minute walk*" OR "6 minute walk*" OR 6MW* OR "walk test" OR "stress test " OR borg* OR SatO2) | 255,22 |
| 6 | 4 OR 5 | 280,475 |
| 7 | 3 AND 6 | 86 |

**Cinahl search strategy:** February 1, 2024

| 1 | (MH "Post-Acute COVID-19 Syndrome") | 1,159 |
| --- | --- | --- |
| 2 | TI ( ("long-term covid*" OR "long covid*" OR "long-covid" OR"chronic covid*" OR "post-acute covid*" OR post-covid* OR "post-acute corona*") OR AB ( ("long-term covid*" OR "long covid*"OR "long-covid" OR "chronic covid*" OR "post-acute covid*" OR post-covid* OR "post-acute corona*") ) OR MW ( ("long-term covid*" OR "long covid*" OR "long-covid" OR "chronic covid*" OR "post-acute covid*" OR post-covid* OR "post-acute corona*") ) | 2138 |
| 3 | 1 OR 2 | 2138 |
| 4 | (MH "Physical Fitness+") OR (MH "Spirometry") OR (MH "Exercise Test+") OR (MH "Exercise Test, Cardiopulmonary") OR (MH "Walking") OR (MH "Respiratory Function Tests") OR (MH "Oxygen Saturation") OR (MH "Oxygen Consumption") OR (MH "Lung Volume Measurements") OR (MH "Spirometry") OR (MH "Respiratory Airflow") | 92531 |
| 5 | TI ("physical fitness" OR "physical endurance" OR "aerobic endurance" OR "physical functional performance" OR "physical function" or "cardiorespiratory fitness" OR "physical exertion" OR exercise OR "cardiorespiratory function" OR "cardiopulmonary function" OR "pulmonary function" OR "lung function" OR "respiratory function" OR spiromet* OR "diffusing capacity for carbon monoxide" OR DLCO OR vo2max OR "maxim* oxygen consumption" OR "oxygen consumption" OR "maxim* oxygen uptake" OR "peak VO2" OR VO2* OR "6-minute walk*" OR "6 minute walk*" OR 6MW* OR "walk test" OR "stress test " OR borg* OR SatO2) OR AB ("physical fitness" OR "physical endurance" OR "aerobic endurance" OR "physical functional performance" OR "physical function" or "cardiorespiratory fitness" OR "physical exertion" OR exercise OR "cardiorespiratory function" OR "cardiopulmonary function" OR "pulmonary function" OR "lung function" OR "respiratory function" OR spiromet* OR "diffusing capacity for carbon monoxide" OR DLCO OR vo2max OR "maxim* oxygen consumption" OR "oxygen consumption" OR "maxim* oxygen uptake" OR "peak VO2" OR VO2* OR "6-minute walk*" OR "6 minute walk*" OR 6MW* OR "walk test" OR "stress test " OR borg* OR SatO2) OR MW ("physical fitness" OR "physical endurance" OR "aerobic endurance" OR "physical functional performance" OR "physical function" or "cardiorespiratory fitness" OR "physical exertion" OR exercise OR "cardiorespiratory function" OR "cardiopulmonary function" OR "pulmonary function" OR "lung function" OR "respiratory function" OR spiromet* OR "diffusing capacity for carbon monoxide" OR DLCO OR vo2max OR "maxim* oxygen consumption" OR "oxygen consumption" OR "maxim* oxygen uptake" OR "peak VO2" OR VO2* OR "6-minute walk*" OR "6 minute walk*" OR 6MW* OR "walk test" OR "stress test " OR borg* OR SatO2) | 267596 |
| 6 | 4 OR 5 | 293919 |
| 7 | 3 AND 6 | 215 |

**Embase search strategy:** August 10, 2022

| 1 | (((covid or corona*) adj1 (long-term or chronic or post-acute or long)) or post-covid or long-covid).ti,ab,kw. | 11612 |
| --- | --- | --- |
| 2 | exp long COVID/ | 1826 |
| 3 | 1 OR 2 | 11896 |
| 4 | ("physical fitness" or "physical endurance" or "aerobic endurance" or "physical functional performance" or "physical function" or "cardiorespiratory fitness" or "physical exertion" or "exercise tolerance" or "exercise test" or ((cardiorespiratory or cardiopulmonary or pulmonary or lung or respiratory) adj1 function) or spiromet* or "diffusing capacity for carbon monoxide" or DLCO or vo2max or "maxim* oxygen consumption" or "oxygen consumption" or "maxim* oxygen uptake" or "peak VO2" or VO2* or "6-minute walk*" or "6 minute walk*" or 6MW* or "walk test" or "stress test" or borg* or SatO2).ti,ab,kw. | 345629 |
| 5 | exp Fitness/ OR exp spirometry/ OR exp Exercise Test/ OR exp Walk Test/ OR exp Exercise Tolerance/ OR exp Exercise/ OR exp Cardiorespiratory Fitness/ OR exp/ lung function test/ OR exp endurance/ OR exp “physical activity, capacity and performance”/ | 1259682 |
| 6 | 4 OR 5 | 1413654 |
| 7 | 3 AND 6 | 1543 |

**Embase search strategy:** February 1, 2024

| 1 | (covid:ti,ab,kw OR corona*:ti,ab,kw) AND adj1:ti,ab,kw AND ('long term':ti,ab,kw OR chronic:ti,ab,kw OR 'post acute':ti,ab,kw OR long:ti,ab,kw) OR 'post covid':ti,ab,kw OR 'long covid':ti,ab,kw | 16350 |
| --- | --- | --- |
| 2 | long covid/'/exp | 6737 |
| 3 | 1 OR 2 | 17480 |
| 4 | physical fitness':ti,ab,kw OR 'physical endurance':ti,ab,kw OR 'aerobic endurance':ti,ab,kw OR 'physical functional performance':ti,ab,kw OR 'physical function':ti,ab,kw OR 'cardiorespiratory fitness':ti,ab,kw OR 'physical exertion':ti,ab,kw OR 'exercise tolerance':ti,ab,kw OR 'exercise test':ti,ab,kw OR ((cardiorespiratory:ti,ab,kw OR cardiopulmonary:ti,ab,kw OR pulmonary:ti,ab,kw OR lung:ti,ab,kw OR respiratory:ti,ab,kw) AND adj1:ti,ab,kw AND function:ti,ab,kw) OR spiromet*:ti,ab,kw OR 'diffusing capacity for carbon monoxide':ti,ab,kw OR dlco:ti,ab,kw OR vo2max:ti,ab,kw OR 'maxim* oxygen consumption':ti,ab,kw OR 'oxygen consumption':ti,ab,kw OR 'maxim* oxygen uptake':ti,ab,kw OR 'peak vo2':ti,ab,kw OR vo2*:ti,ab,kw OR '6-minute walk*':ti,ab,kw OR '6 minute walk*':ti,ab,kw OR 6mw*:ti,ab,kw OR 'walk test':ti,ab,kw OR 'stress test':ti,ab,kw OR borg*:ti,ab,kw OR sato2:ti,ab,kw | 268851 |
| 5 | fitness'/exp OR 'spirometry'/exp OR 'exercise test'/exp OR 'walk test'/exp OR 'exercise tolerance'/exp OR 'exercise'/exp OR 'cardiorespiratory fitness'/exp OR 'lung function test'/exp OR 'endurance'/exp OR 'physical activity, capacity and performance'/exp | 1447206 |
| 6 | 4 OR 5 | 1542128 |
| 7 | 3 AND 6 | 2483 |
